# Ylehd, an epoxide hydrolase with promiscuous haloalkane dehalogenase activity from tropical marine yeast *Yarrowia lipolytica* is induced upon xenobiotic stress

**DOI:** 10.1038/s41598-017-12284-9

**Published:** 2017-09-19

**Authors:** Chandrika Bendigiri, Smita Zinjarde, Ameeta RaviKumar

**Affiliations:** 1 Institute of Bioinformatics and Biotechnology, Savitribai Phule Pune University, Pune, 411007 India; 2 Department of Biotechnology, Savitribai Phule Pune University, Pune, 411007 India

## Abstract

Recalcitrant environmental pollutants, like bromoorganics and epoxides are hydrolysed with limited substrate specificities by microbial oxygenases, reductases, hydrolases and dehalogenases. Here, we report the identification and characterisation of a protein (XP_504164) from the tropical marine yeast *Yarrowia lipolytica* NCIM 3589, known to degrade bromoorganics and epoxides. Multiple sequence alignment suggests it belongs to α/β superfamily with conservation of catalytic triad and oxyanion hole motifs. The corresponding gene cloned and protein (Ylehd) expressed in *E. coli* BL21AI exhibited epoxide hydrolase activity (24 ± 0.7 nmol s^−1^ mg^−1^ protein) at pH 8.0 and promiscuous haloalkane dehalogenase (1.5 ± 0.2 nmol s^−1^ mg^−1^ protein) at pH 4.5. Recombinant Ylehd catalyses structurally diverse epoxides and bromoorganics with maximum catalytic efficiency (k_cat_/K_m_) of 96.56 and 10.1 mM^−1^ s^−1^ towards 1,2-Epoxyoctane (EO) and 1-Bromodecane (BD). The expression of Ylehd was highly induced in presence of BD and EO but not in glucose grown cells as studied by immunoblot analyses, q-PCR and activity levels. Immunoelectron microscopy confirmed higher expression in presence of xenobiotics and located it to cytosol. Such inducible nature of Ylehd suggests its physiological role in xenobiotic stress mitigation. This study represents the first functional characterisation of a bifunctional EH/HLD in eukaryotic microbes with broad substrate specificity making it a potential biocatalyst for bioremediation/biosensing of mixed pollutants.

## Introduction

The widespread use of industrially important organobromines and epoxides in pesticides, disinfectants, flame retardants, pharmaceuticals and polymers has led to their build up in the environment. Many of these chemicals or their metabolic dead-end products are highly recalcitrant and persistent, accumulating in water, sediments, aquatic and terrestrial life forms^1^. Therefore,the effects on ecosystem of these brominated and aromatic hydrocarbons which degrade to mutagenic epoxides having differing reactivities and potencies^2^ are of major concern. Conventional treatment technologies such as landfills, ‘pump and treat’, high-temperature incineration and chemical decomposition (by base-catalyzed dechlorination, UV oxidation) have many drawbacks. These methods are time consuming, invasive, disruptive to natural habitats and results in rearrangement of the problem. For example, in landfills, the contaminations remain and can leach and pollute the surrounding soil and ground water. For less developed countries, incineration is technologically complex and expensive while chemical decomposition often employ hazardous chemicals that create additional environmental risks. Hence, bioremediation, employing biological processes such as microbes for degradation and removal of xenobiotics offers a better solution as it is safe, simple, efficient, economical and eco-friendly as it can reduce the levels of these pollutants to acceptable limits. The main challenge is to identify microbes with appropriate enzymes that can detoxify contaminants by transformation or degradation. Also, effective use of microbial bioremediation is currently hampered by an incomplete understanding of metabolic pathways, the enzymes involved and their kinetics^3^. Therefore, there is a dire need to overcome these deficiencies and make bioremediation processes eco-friendly and cheap.

Marine biotas are known to produce a *plethora* of brominated compounds that serve as chemical defence against predators and in biofouling^4^. Organobromines and epoxy compounds of anthropogenic origin have also been deposited in oceans over a period of time, compelling marine microbes to adapt to the harsh environmental conditions. In fact, microflora have been reported that can degrade organohalides, epoxides and their derivatives, by employing novel metabolic pathways regulated by enzymes such as dehalogenases and epoxide hydrolases^5–7^. Dehalogenases cleave the carbon-halogen bond by different mechanisms, namely, reductive, thiolytic, oxidative and hydrolytic^5^. Haloalkane dehalogenases (HLD, E.C 3.8.1.5) have been well studied in bacteria and catalyze the hydrolytic breakdown of aliphatic halogenated compounds to alcohols and halides^8^. Epoxide hydrolase (EH, EC 3.3.2.3) are versatile enzymes that catalyse the addition of water to the reactive epoxides or aromatic hydrocarbons and detoxify them to diols^9^. Both these enzyme belong to the α/β hydrolase superfamily which are diverse in catalytic activity and are characterised by a low sequence but high degree of structural similarity^10^. Microbes possessing such enzyme activities are thus important in the bioremediation of polluted soil, groundwater, and wastewater. In the above context, the past few years have seen the emergence of non-conventional yeasts in bioremediation of numerous industrial and environmental wastes by employing essential enzymatic makeup to mediate natural detoxification. *Trichosporon* sp., *Candida tropicalis* and *Yarrowia lipolytica* have been effective in reducing chemical oxygen demand up to 95%^11–13^. The Central Pollution Control Board of India has detected the presence of brominated pesticides and flame retardants in industrial effluent belts along the Indian coastal waters of Arabian Sea^14^. These marine waters are polluted by disposal of untreated sewage, industrial effluents, oil tanking depots and port wastes. A tropical marine yeast, *Y. lipolytica* NCIM 3589, isolated from these polluted waters off Mumbai High oil field is known for its ability to degrade hydrophobic substrates, detoxify nitroaromatics, organophosphates and in removal of metal ions^15^. Our previous studies have demonstrated that this yeast is able to utilize and metabolize bromoaliphatics and bromoaromatics as sole source of carbon and energy with concomitant release of bromide^6,16^. No dehalogenase has been reported to date in eukaryotic microbes. In contrast, epoxide hydrolases are ubiquitous and have been widely reported from yeasts^17^.

In the present study, we identify and functionally characterize the protein responsible for dehalogenation in the yeast. In this pursuit, a novel bifunctional enzyme exhibiting epoxide hydrolase (EH) activity at pH 8.0 and a promiscuous haloalkane dehalogenase (HLD) activity at pH 4.5 is being reported for the first time. In the host strain *Y. lipolytica* NCIM 3589, the protein was found to be inducible under physiologically simulated xenobiotic stress conditions. Thus, this robust yeast has the potential to be used for bioremediation of organohalides and epoxides.

## Results and Discussion

### Identification and phylogenetic analysis of gene sequence in *Y. lipolytica* 3589

**Figure 1:**
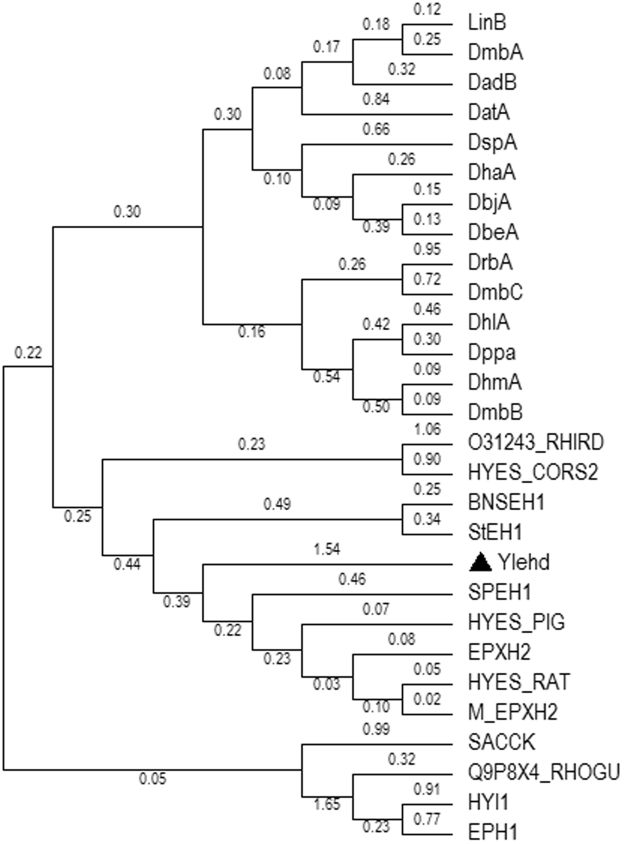
Phylogenetic tree for Ylehd. Sequence information is as given in Table S1. The evolutionary history was inferred by using the Maximum Likelihood method based on the JTT matrix-based model. The tree with the highest log likelihood (−8269.0505) is shown. Initial tree(s) for the heuristic search were obtained automatically by applying Neighbor-Joining and BioNJ algorithms to a matrix of pairwise distances estimated using a JTT model, and then selecting the topology with superior log likelihood value. The analysis involved 15 amino acid sequences. All positions containing gaps and missing data were eliminated. There were a total of 244 positions in the final dataset.

To date, no reports on the functional presence of HLD in microbial eukaryotes are available. Given this situation, structurally and functionally well characterized bacterial HLD sequences were initially aligned so that a consensus sequence could be identified which was searched in *Y. lipolytica* non-redundant NCBI sequence database. Three hits with accession numbers, namely, XP_504164, XP_499652, XP_502171 with similarity scores of 46.6, 43.5 and 40%, respectively were identified as putative EH-like. Since XP_504164 exhibited the best score, studies were initiated with it and was called Ylehd. The ESTHER database, a collection of information on members of the α/βsuperfamily, classified XP_504164 as EH-like^18^ as also theSwiss-Protdatabase with accession Q6C598. It is interesting to note that using the consensus sequence derived from the alignment of HLD sequences, an EH hit was obtained suggesting that these two families of enzymes are closest relatives in the /β hydrolase superfamily. A phylogenetic tree was constructed with twenty seven protein sequences. Epoxide Hydrolase (EH), sequences from representative well characterized proteins (13 numbers) of plant, animal, bacterial and fungal source were considered while for Haloalkane dehalogenase (HLD), well characterized proteins (14 numbers) from bacterial sources and DspA from *Strongylocentrotus purpuratus* were studied. On analysis by using the maximum likelihood method, results indicate that EH and HLD cluster separately (Fig. 1). Ylehd was found to lie on a separate clade along with EH proteinsand is highly diverged and remotely removed from EH sequences of yeast and fungal origin as well as from the HLD sequences characterised to date. It seems to be closely related to EHs from higher eukaryotic forms with the closest resemblance with SPEH1 from the sea urchin *Strongylocentrotus purpuratus* and StEH1 from plant *Solanum tuberosum*. The details of the protein sequences considered for the study are as indicated in Supplementary Table S1.

### Multiple sequence alignment and comparison of Ylehd

**Figure 2:**
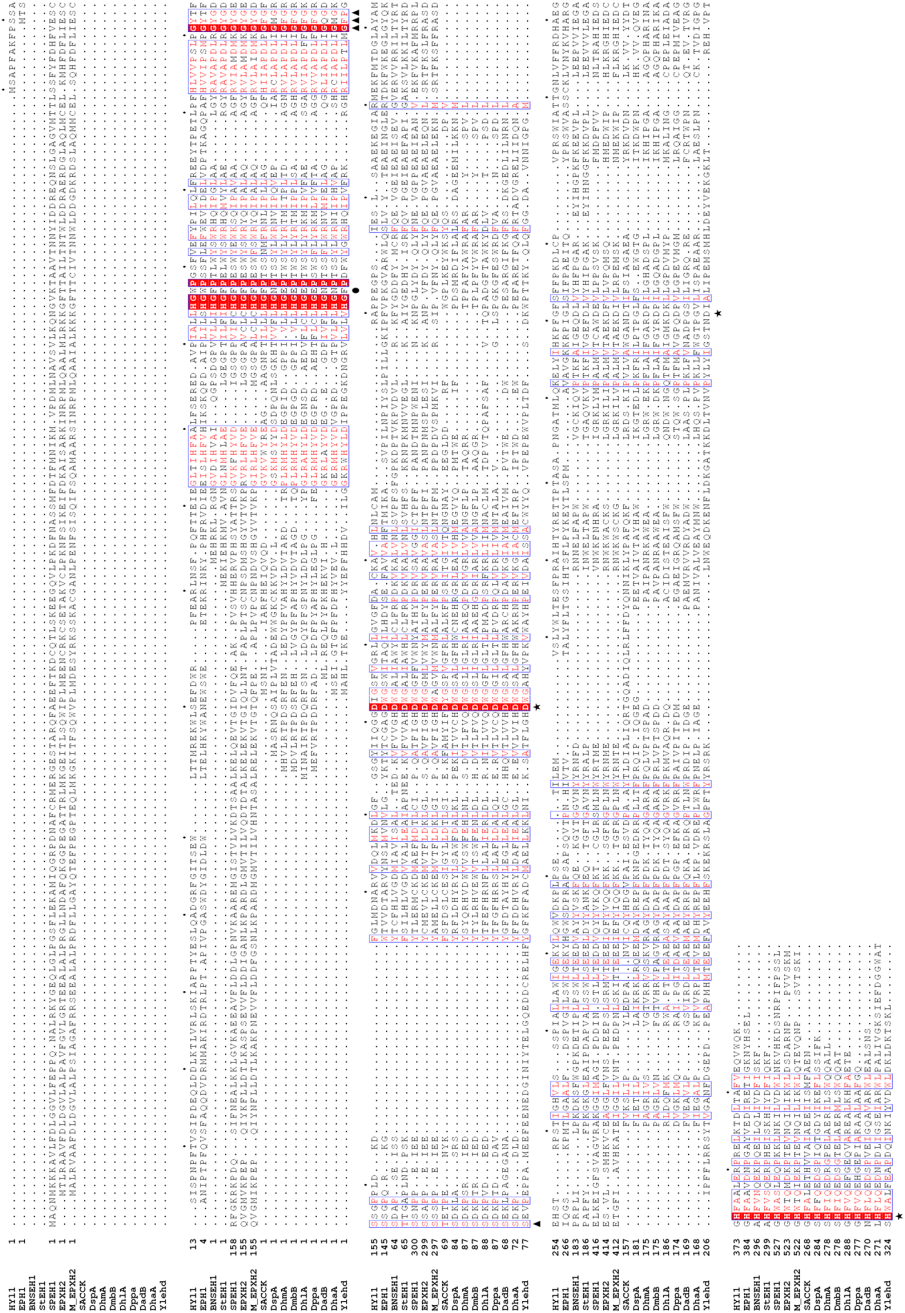
Multiple sequence alignment of Ylehd with EHs and HLDs. Protein sequences considered for the study are as given in Table S1. ★ indicates conserved residues of catalytic triad, ● indicates phenylalanine residue of the HGXP motif. ▲ indicate residues forming the GXSmXS/T. Residues marked in red and yellow indicate identity and similarity respectively.

EHs and HLDs, belonging to the α/β super family have a conserved catalytic triad consisting of a nucleophile, acid and an invariant histidine as base along with a conserved HGXP motif forming the oxyanion hole^19^. Multiple sequence alignment (MSA) for Ylehd with functionally well characterized EHs and HLDs was carried out to determine conservation of aminoacid residues, if any. The results suggest that the conserved catalytic residues were D140 as nucleophile, H325 as invariant base while the acid residue was assigned as D296 (Fig. 2). It also shows conservation of HGXP motif between residues 43 and 46 with X represented by phenylalanine (F). The X residue of the HGXP motif is an aromatic residue in EHs and either N/E in HLDs, and is a key differentiating feature between EH and HLD^20^. Thus, based on the residues in the oxyanion hole (HGFP), Ylehd can be categorized as an EH rather than HLD and shares this motif similarity to EH from *S. cerevisiae* (SACCK), *S. purpuratus* (SPEH1), mammalian soluble EH (sEH) and other EHs as seen in Fig. 2. However, proteins with diverse signature motifs exist, as the oxyanion hole in EH from the fungus *Phanerochaete chrysosporium*, is unique with N instead of an aromatic residue at the X position^21^. Other residues, namely, GFPGS, from 73 to 76 in Ylehd represents the GXSmXS/T (X: any amino acid, Sm: small amino acid) conserved motif in proteins of the α/β hydrolase superfamily.

Furthermore, it is interesting to note in MSA that a large portion of N-terminal region present in EHs is missing in Ylehd similar to that for HLDs from bacterial sources. It is to be noted that the ESTHER database classifies HLDs under EH-like family^18^. Human soluble EH seem to have evolved from gene fusion event between N-terminal domain of haloacid dehalogenase and C-terminal domain of haloalkane dehalogenase with HLDs as an ancestor^22^. Thus, the above analysis suggests that Ylehd most likely belongs to the EH-like family of α/β hydrolase superfamily, with scope for further functional characterization. Hence, studies were initiated to determine if Ylehd functionally exhibited EH and/or HLD activity.

### Cloning, expression and purification of Ylehd

Codon usage of *Y. lipolytica* was checked at http://gcua.schoedl.de which indicated that *E. coli* was a suitable host for protein expression. The *ylehd* gene was cloned into pET28a vector which upon sequencing, coded for a 352 amino acid polypeptide with mutation of non–conserved E88G attributed to strain variation between the natural isolate under study (*Y. lipolytica* NCIM 3589) and the one reported in NCBI database (*Y. liplolytica* CLIB122). The recombinant His-tagged Ylehd was expressed in *E. coli* BL21AI and the lysate loaded on a Ni-NTA column with a yield of 20 mgL^−1^ protein. Further purification on size exclusion chromatography showed that the protein eluted as a single peak. A single band was seen on native-PAGE as well as on SDS-PAGE with a M_r_ of 43 kDa (Supplementary Fig. S1a,b).

### Biochemical characterization of Ylehd

To obtain insight into its functionality and physico-chemical properties, the HLD and EH activities of Ylehd were determined using 1-Bromodecane (BD) and 1,2-Epoxyoctane (EO) as substrates, respectively. BD was the substrate of choice based on our earlier studies with the parent strain *Y. lipolytica* NCIM 3589^16^ while EO is routinely used for screening of yeast EH^17^.

#### Epoxide Hydrolase activity and product identification

**Figure 3:**
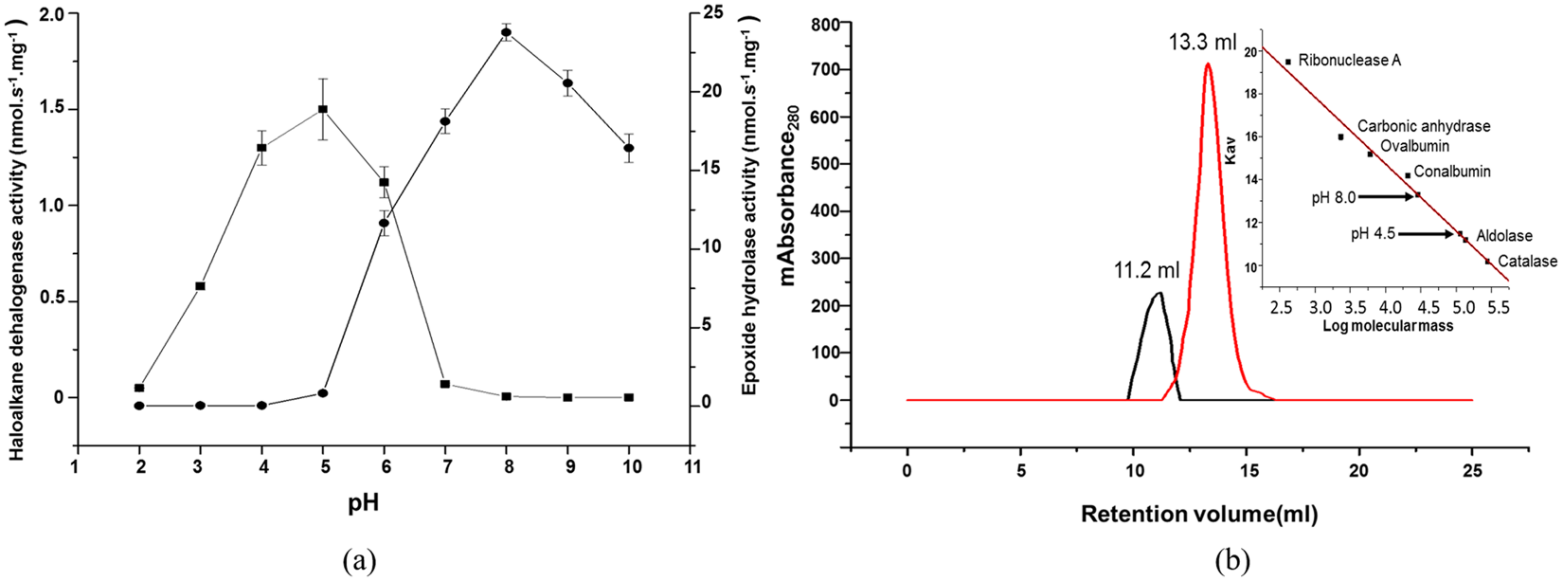
Optimum pH and size exclusion chromatography for Ylehd. Specific activity for EH (■) and HLD (●) activity of Ylehd at pH 8.0 and 4.5. pH dependent oligomerization as seen in size exclusion chromatography of Ylehd on Sephadex S200 column in 50 mM sodium acetate pH 4.5 (black) and 50 mM Tris-sulphate pH 8.0 (red) Inset: Determination of molecular mass using proteins of known molecular mass as standards (kDa) Ribonuclease A-13.7, Carbonic anhydrase-29, Ovalbumin-43, Conalbumin-75, Aldolase-158, Catalase-23.

Using Britton-Robinson buffers, the optimal pH for EH activity was found to be 8.0 with maximal activity of 24 ± 0.7 nmol s^−1^ mg^−1^ protein. More than 90% loss in activity was seen at pH 5 and below under the given assay conditions (Fig. 3a). This was similar to other EHs studied wherein a pH 6.0–8.0 was found to be optimal for enzyme function. Overlay of the native gel after electrophoresis on EO emulsified agar plate showed a clearance zone suggesting hydrolysis of EO present in the agar by Ylehd (Supplementary Fig. S1c).

**Figure 4:**
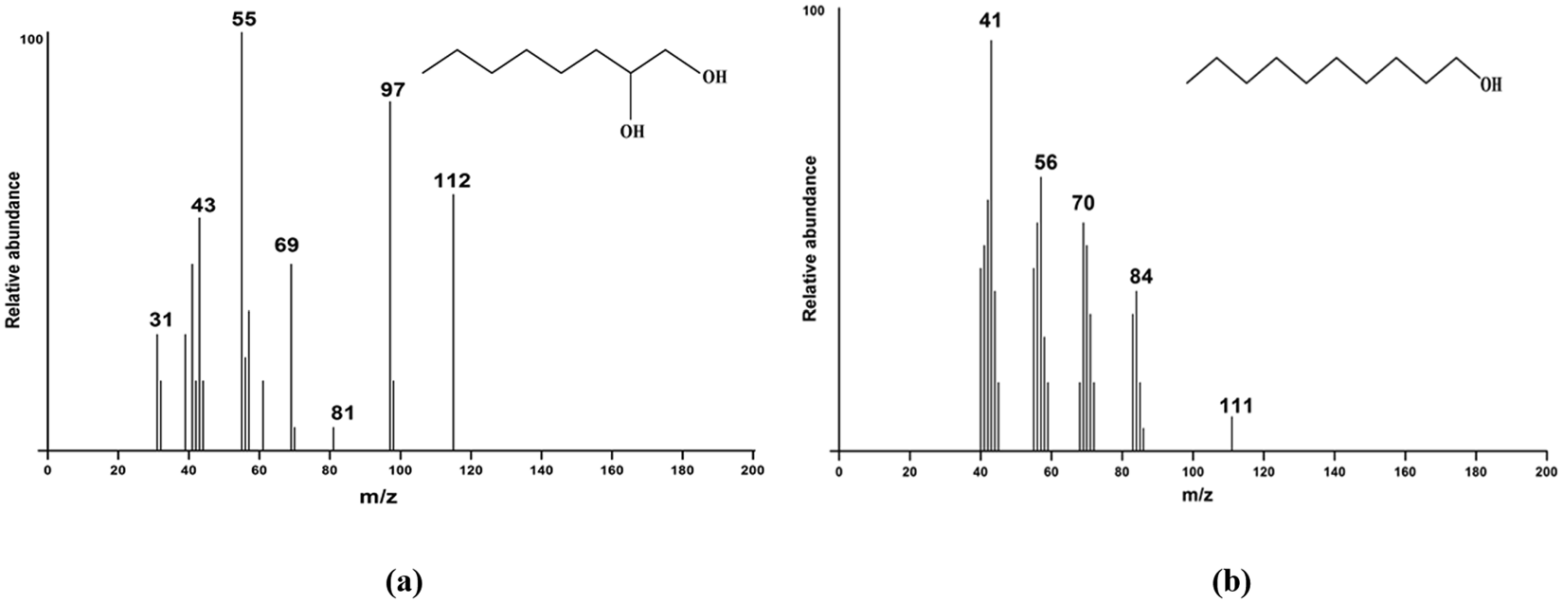
Product identification. GC-MS spectrum and structure of (**b**) 1,2-Octanediol and (**c**) 1-Decanol.

Product identification of EH reaction at pH 8.0 using EO as substrate was carried out by GC-MS. The product eluted at RT = 8.98 min with m/z = 146 and exhibited daughter fragments of m/z (M^−^ H^+^) 31, 43, 55, 69, 81, 97, 112 (Fig. 4a). The best hits obtained were for 1, 2-octanediol with the NIST Mass Spectral Library (NIST 05), confirming EH activity.

#### Dehalogenase activity and product identification

The ability of *Y. lipolytica* 3589 to use bromoalkanes as sole source of carbon and energy with the release of bromide, required a dehalogenating enzyme and could not be explained by EH activity. Using BD as substrate, the optimal pH for HLD activity was found to be 4.5 with a specific activity of1.5 ± 0.2 nmol s^−1^ mg^−1^ protein. The enzyme lost >90% of its activity in the alkaline pH range (Fig. 3a). Zymogram of the gel after incubation with BD as substrate showed the presence of a grey coloured band (Supplementary Fig. S1d).

Bioconversion of BD carried out under nitrogen purged conditions to remove interference by aerial and dissolved oxygen resulted in the formation of a product eluting at RT = 17.78 min. GC-MS analysis showed that it had a molecular mass of m/z = 157 and exhibited daughter fragments at the m/z (M^−^ H^+^), 41, 56, 70, 84 and 111 similar to that of standard 1-decanol. Also the best hits obtained were for 1-decanol with the NIST Mass Spectral Library (NIST 05), Thus, hydrolytic cleavageof the carbon-halogen bondwhichoccurred under oxygen free conditions, resulted in the formation of 1-decanol confirming HLD activity of Ylehd at pH 4.5 (Fig. 4b).

An optimal pH of 4.5 for HLD seems unusual and is being reported for the first time as all other HLDs reported till date from bacterial sources have an optimal pH between 6–9 though Dumb from *Mycobacterium bovis* has two pH optima at 5.5 and 9.0^23^. Thus, it can be seen that Ylehd showed pH dependent dual activity *viz*., EH at pH 8.0 and HLD at pH 4.5. Dual activity for EH is reported in the case of soluble mammalian EH (sEH) wherein a phosphatase domain is seen at the N-terminal end of the protein while Leukotriene A_4_ hydrolase is another bifunctional enzyme with EH and aminopeptidase activity^9,24^. So far for HLD, only a single report exists of a tautomerase with a promiscuous dehalogenase activity in *Mycobacterium* sp^25^. Such promiscuous/bifunctional behaviour of enzymes and their functional diversity are not unusual and could arise due to presence of two or more functional domains and/or environment induced conformational changes in the protein resulting in unique evolutionary features^26^. To the best of our knowledge, no reports of a bifunctional EH/HLD activity exist to date in biological systems.

Further, no phosphatase activity was detected for Ylehd when tested with p-nitrophenyl phosphate as substrate, unlike the mammalian sEH. This is not surprising as the N-terminal domain for sEH having phosphatase activity has arisen from haloacid dehalogenase while MSA analysis shows that this N-terminal region missing in Ylehd. Also, no lipase, esterase, aminopeptidase, cytochrome P450 monooxygenase and reductaseactivities could be detected for this recombinant protein. In this study, we report for the first time a bifunctional enzyme with EH at pH 8.0 and HLD activity at pH 4.5 from the tropical marine yeast *Y. lipolytica* NCIM 3589.

The optimum temperature of activity for both EH and HLD activity was found to be 30 °C (Supplementary Fig. S2).

#### pH induced changes in Ylehd

In order to obtain insights into its pH dependent structural aspects, the far-UV CD spectrum was determined and analysed using SELCON 3 program from Dichroweb. On shift from pH 8.0 to 4.5, the CD spectra underwent a change (Supplementary Fig. S3). The CD spectra at pH 8.0 was indicative of a spectra similar to EHs from α/β superfamily with 37% α-helices and 16% β-strand. At pH 4.5, the α-helical content decreased to 22% whilethe β-strand content increasedto 31%. No change in the content of random coils (29%) and turns (18–20%) was seen when the pH changed from 8.0 to 4.5. This implies that the conformational transition from α-helical to β-strands on pH shift may play a role in determining the function of Ylehd. The α-helical and β-strand content for EH from *Agrobacterium radiobacter* AD1(EchA) were 28 and 22%^27^ while that from human and murine liver were 39 and 38%, respectively^28^, similar to Ylehd. In contrast to Ylehd, the α-helical content in bacterial HLDs was found to be higher in LinB (40.3%), DhaA (43%), DhlA (40.9%) and DbjA (60%)^29^.

Further, on characterizing the protein by size exclusion chromatography, showed that at pH 8.0 Ylehd eluted at 13.3 ml while it eluted at 11.3 ml at pH 4.5, with calculated molecular weights of 86 kDa (K_av_ = 0.3) and 170 kDa (K_av_ = 0.173) as seen in Fig. 3b. The calculated Stokes radius of the protein increased from 5.16 to 6.63 nm when the pH was changed from 8.0 to 4.5. Thus, change in elution profile, increase in molecular weight and Stokes radius suggest a pH dependent oligomerization of the protein which in turn seems to be affecting its functionality. Considering the monomeric mass of the protein to be 43 kDa as seen by SDS-PAGE, it is likely that Ylehd exists as a dimer at pH 8.0 and as a tetramer in pH 4.5.

It is to be noted that different oligomeric forms have been reported for EH and HLD proteins, wherein a correlation between the oligomeric state of the protein and activity has been established. Other fungal EHs known to exist as homodimers with apparent molecular weights of 66.8 kDa for *Saccharomyces cerevisiae* and 79 kDa for *Apsergillus niger* and *Aspergillus brasiliensis* CCT1435 (AbEH)^30,31^. In fact, mammalian sEH is a dimer with Mr 64 kDa and dimerization is essential for its activity which could also be the case for fungal EHs. In contrast, the EH from *Agrobacterium radiobacter* and *Brassica nappus* were found to be catalytically active as monomer with molecular mass of 34 and 37 kDa, respectively suggesting that different oligomeric states could be involved in the functionality of EH proteins^27,32^.

In the case of HLDs, a strong pH dependent oligomerization has also been observed. For example, DbjA *from Bradyrhizobium japonicum*, active towards β-bromoalkanes at pH 9, exists as a monomer at pH 5.9, while the catalytically active dimeric, tetrameric and higher oligomeric states are predominant at pH 6.1, 9.6 and 10.2, respectively^33^.

DadB from marine bacterium *Alcanivorax dieselolei* B-5, oligomerizes in buffers with pH ≤ 7.0 while DrbA from *Rhodopirrellula baltica* and DmbC from *Mycobacterium bovis* 5033/66 are proteins wherein oligomerization has been observed with sizes >2,000 kDa with catalaytic activity seen^34,35^.

Thus, it is likely that in Ylehd, a change in the oligomeric state as well as rearrangement of the secondary structureinduced on pH switch could be major segregating factors for the two enzyme activities.

#### Substrate specificity for Ylehd

**Figure 5:**
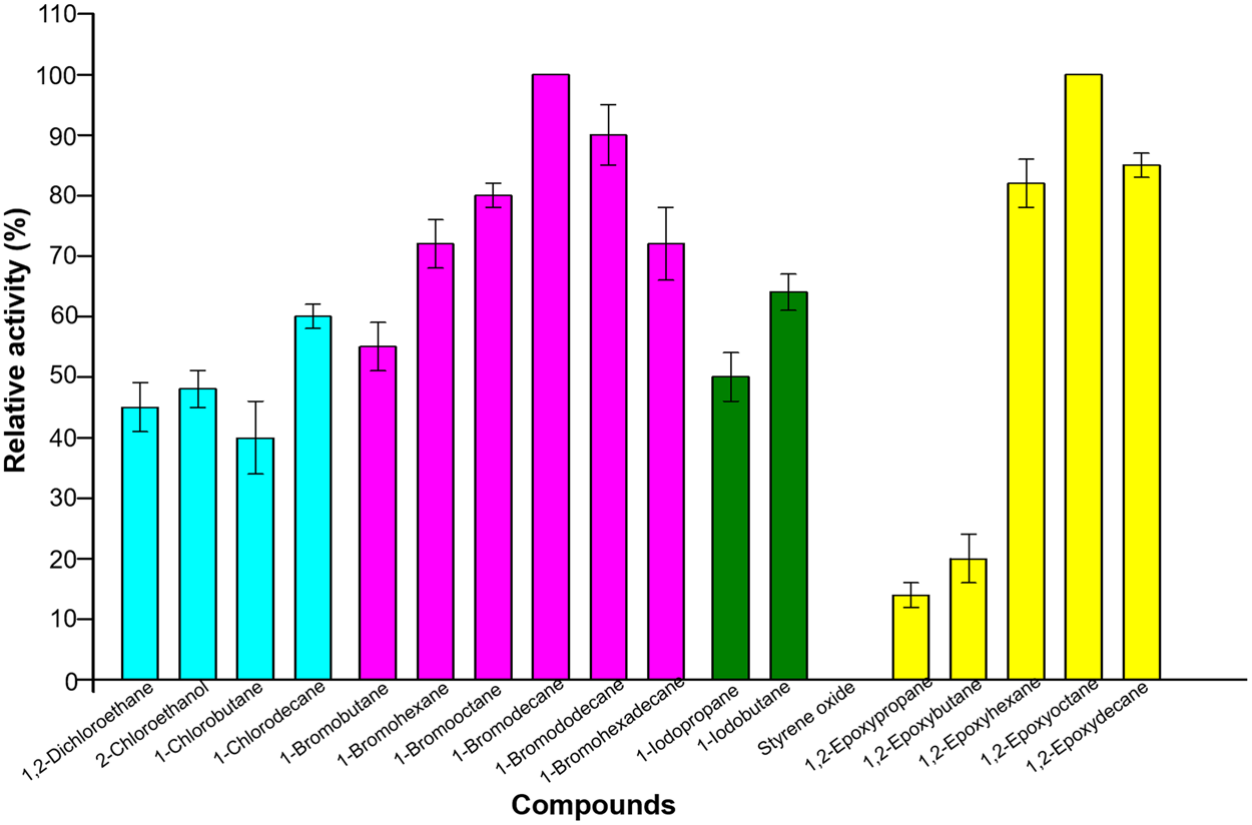
Substrate specificity for EH and HLD activity of Ylehd. Substrate specificity of Ylehd tested with halogenated compounds and epoxides with different scaffolds. All values expressed as percent relative activity with respect toBD among halogenated compounds and EO for epoxides.

Enzyme activities for both HLD and EH function of Ylehd were investigated towards compounds with differing scaffolds (Fig. 5). HLD was found to exhibit broad substrate specificity towards halogenated compounds with relative activity ranging between 40–100%. It was mainly active towards monosubstituted short and medium chain length (C2-C16) bromoalkanes with highest specific activity towards BD (1.5 ± 0.2 nmol s^−1^ mg^−1^) at pH 4.5 while lower dehalogenase activity was seen towards iodinated and chlorinated alkanes. HLD proteins are known to exhibit a broad substrate specificity^8^. DspA from the sea urchin *S. purpuratus* ^36^ and DadB from *A. dieselolei* B-5^34^ both from marine environment also exhibited a preference for brominated compounds, likely for organisms found in that niche. DbjA from *Bradyrhizobium japonicum*, DhlA from *Xanthobacter autotrophicus* and DhaA from *Rhodococcus* sp. strain NCIMB13064 exhibit activity towards a wide variety of chloro, bromo and iodo compounds including 1,2 dichloroethane. It has been suggested that the variation seen in the substrate specificity of proteins may arise from the flexible movement of the residues lining the tunnel leading to the active site cavity^8^.

Maximal EH specific activity was exhibited for EO (24 ± 0.7 nmol s^−1^ mg^−1^) at pH 8.0. Lower EH activity was seen towards 1,2-epoxypropane <1,2-epoxybutane <1,2-epoxyhexane and 1,2-epoxydecane while none could be detected towards styrene oxide even up to 5 mM of substrate concentration and 20 μg of Ylehd. It is to be noted that EH from *A. radiobacter* which shares only 24.9% sequence identity with Ylehd is also found to be a broad substrate specific enzyme with activity towards 1,2-epoxybutane and 1,2-epoxyoctane. An interesting feature is that though this protein from *A. radiobacter* aligns completely with the catalytic nucleophile and base-histidine of the bacterial HLDs, no HLD activity is reported for this protein unlike Ylehd^27^. Yeasts such as *Rhodotorulla glutinis* and *Rhodosporidium toruloides* showed similar epoxide specificity as Ylehd i.e., with preference towards 1,2 epoxides of chain lengths C6-C8 rather than towards styrene oxide and p-nitro styrene oxide^17^. In contrast, EHs from fungi like *Aspergillus niger* were found to be more specific towards styrene oxides^37^ while PchEHA from *P. chrysosporium* could act on epoxides with varied structural scaffolds such as styrene oxide and 1,2-epoxybutane^21^.

#### Kinetic properties for Ylehd

The kinetic parameters, K_m_ and k_cat,_ were compared for some of the substrates from each class of compounds, namely, epoxides and haloalkanes (Table 1). The recombinant protein showed lower K_m_ values towards BD (0.01 mM) than towards EO (0.5 mM) indicating higher affinity towards bromoalkanes. However, the catalytic constant (k_cat_) was found to be higher for EO (48.28 s^−1^) than BD (0.1 s^−1^) suggesting a higher turnover for EO. The specificity constant, k_cat_/K_m_ is several orders of magnitude greater for EH (96.5 mM^−1^ s^−1^) than HLD (10.11 mM^−1^ s^−1^) indicating that Ylehd has a greater catalytic efficiency towards epoxides. This suggests that the protein is an EH with promiscuous HLD activity. Likewise, in human sEH^22^, the catalytic efficiency (k_cat_ */*K_m_) of the EH domain is more (2.56 × 10^6^ M^−1^ s^−1^) than the phosphatase domain (1.67 × 10^4^ M^−1^ s^−1^). Similar to Ylehd, the dehalogenases DrbA and DmbC from *Mycobacterium* exhibited low K_m_ values of 0.063 and 0.018 mM towards1-iodobutane suggesting a high affinity of the enzyme towards it while k_cat_ of 0.128 and 0.0715 s^−1^ indicate a low conversion rate with k_cat_/K_m_ values of 2.03 and 3.97 mM^−1^ s^−1^, respectively^35^. The HLDs from marine organisms *S. purpuratus* (DspA) and *A. dieselolei* (DadB) had high affinity (K_m_) of 0.92 and 0.82 mM and k_cat_/K_m_ values of 1.71 and 16.43 mM^−1^ s^−1^, respectively for 1,3-dibromopropane^4,23^.

**Table 1:** Kinetic properties of Ylehd.

Compound	V_max_ (μM min^−1^)	K_m_ mM	k_cat_^a^ s^−1^	k_cat_/K_m_ mM^−1^ s^−1^	Structure
1,2-Epoxyhexane	0.18 ± 0.006	16.37 ± 0.97	32.78 ± 1.33	2.0 ± 0.05	
1,2-Epoxyoctane	0.133 ± 0.02	0.5 ± 0.045	48.28 ± 1.98	96.56 ± 1.55	
1,2-Epoxydecane	0.12 ± 0.05	6.88 ± 0.78	46.33 ± 1.56	6.73 ± 0.82	
2-Bromopropane	5.12 ± 0.45	1.57 ± 0.12	0.74 ± 0.04	2.12 ± 0.56	
1-Bromodecane	4.15 ± 0.87	0.1 ± 0.008	0.1 ± 0.005	10 ± 0.45	
1-Bromododecane	1.24 ± 0.06	0.16 ± 0.05	0.018 ± 0.005	8.8 ± 0.97	

^a^Kinetic studies were carried out with 2 μg protein for EH activity and with 20 μg of purified Ylehd for HLD activity.

The results for Ylehd suggest that while the affinity of substrates is higher towards HLD, the conversion of substrates is greater for EH. It is likely that Ylehd from the yeast with potential for debromination due to its presence in the marine niche could have evolved its EH activity for adaptation to the diverse stress conditions from natural and anthropogenic sources.

#### Effect of additives

The recombinant protein was also tested for activity in the presence of additives such as glycerol, reducing agents, EDTA and metal ions. Glycerol was found to stabilize the enzyme activity and was included in all steps during purification. EDTA (up to 5 mM) did not affect either HLD or EH activity. β-mercaptoethanol, DTT and reduced glutathione selectively increased HLD activity upto 2-fold while no effect was seen on EH (Supplementary Table S2). Such an incremental effect of reducing agents has not yet been reported for any of the known HLD proteins. In contrast, the HLD activity from *Mycobacterium bovis* (DhmA) decreased in the presence of β-mercaptoethanol and EDTA^38^. DTT, β-Mercaptoethanol and reduced glutathione are used as redox reagents to prevent formation of disulfide bonds in cysteine-containing proteins which react stoichiometrically with sulfhydryl reagents resulting in alteration of enzymatic activity. Addition of reducing agents to the protein purification protocols is a routine procedure and is reported in the case of several haloalkane dehalogenase enzymes such as in case of Dhla from *Xanthobacter autotrophicus* ^39^, DmrA and DmrB from *Mycobacterium* strain JS60^40^. Further it is also to be noted that in α/β-hydrolase proteins the nucleophile residue could be either Ser/Cys/Asp^19^. From the sequence analysis it can be seen that Ylehd has seven cysteine residues in the monomer and hence the reducing agents could be affecting enzyme activity.

Metal salts (1–5 mM) had varying effects on HLD and EH activity of Ylehd. Both EH and HLD activities were strongly inhibited by Ag^2+^, Hg^2+^, Pb^2+^, Cu^2+^, Mn^2+^ and Co^2+^ salts while Fe^3+^, Fe^2+^, Ni^2+^ and Zn^2+^ completely inhibited HLD activity but had relatively less effect on EH activity. On the other hand, Mg^2+^ had no effect on eitherfunctions under the given assay conditions. The EH (PchEHA) from white-rot fungus *P. chrysosporium* is not sensitive to EDTA, and is inhibited by several metal ions including Zn^2+^, Cd^2+^ and Hg^2+^ ^21^. Similar to Ylehd, a high degree of inhibition up to 83, 93 and 89% has also been reported with Fe, Ni and Zn salts, respectively for haloalkane dehalogenase isolated from *Rhodococcuserythropolis* Y2^41^. No explanation in the reports has been given for the loss in activity for either *P. chrysosporium*is or *R. erythropolis* Y2. Though being an important cofactor, Zn is also known to interact with free thiol groups causing inhibition as in the case of a fungal dehydrogenase namely 6-Phosphogluconate dehydrogenase while Ni is well-known inhibitor of alpha KG- dependent hydroxylases, yeast hexokinase and horseradish peroxidase though the underlying mechanisms are poorly understood^42^. *S. cerevisae* glutathione reductase was inhibited with Ni competitively while Zn was found to be non-competitively inhibit the enzyme affecting its structure. It has been suggested that interaction of metal ions with the aminoacids in the active site pocket. could be affecting the p*Ka*s of the residues^43^.

Thus this significant differential effect of metal ions on EH and HLD activity at varying pH of Ylehd could find applications in biocatalysis and bioremediation of contaminated sites.

### Intracellular Ylehd expression levels in yeast

To ascertain the role of Ylehd under xenobiotic stress, its intracellular levels were determined under simulated marine conditions. For this, cells were grown on three substrates, namely, glucose as control and on BD and EO as model xenobiotics in sea salts medium.

#### Immunoblot analysis

**Figure 6:**
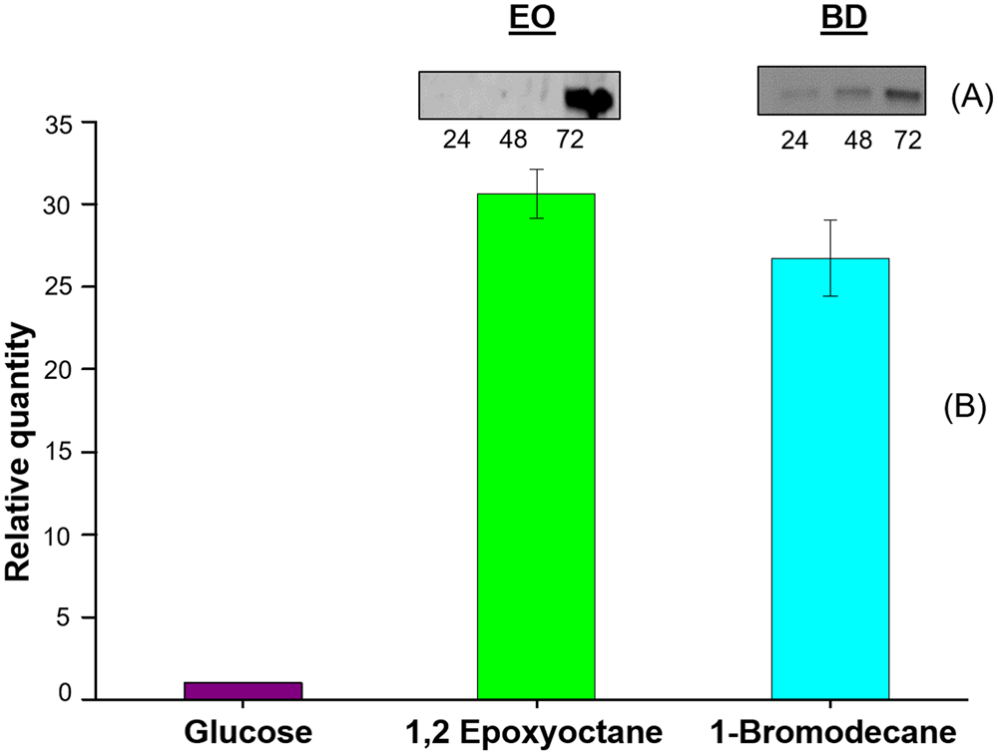
Intracellular expression of Ylehd. Lysates prepared from cells grown in Glucose, EO and BD were lysed at specific time points and analysed by. (**A**) Western blotting and immunodetection (cropped images). (**B**)q-PCR analysis: Normalization for estimation of relative quantity was done with respect to values obtained for glucose grown cells.

Cross-reactivity was seen between the polyclonal antibodies raised in rabbits against Ylehd. A time course study to check the intracellular Ylehd levels was carried out in the yeast cellsusing Western blot followed by immunodetection as mentioned in methods. No expression of Ylehd was seen even at 72 h in glucose grown cells. On the other hand, yeast cells grown on BD exhibited expression at 24 and 48 h reaching a maximum at 72 h while EO grown cells also showed expression at 72 h as seen in the cropped images in Fig. 6A. The full length blots and additional blots showing faint bands seen at 24 and 48 h are shown in Supplementary Fig. S4. Results thus indicate that expression of Ylehd isolated from this strain is substrate inducible and occurs in presence of EO and BD but not in glucose.

Inducible HLD expression in presence of haloalkanes has been seen in *Rhodococcus rhodochrous* ^44^ and for DmrA from *Mycobacterium* strain JS60^40^ while EH from soybean is found to be ethylene inducible^45^ and that from *Trichosporon loubierii* ECU1040 was induced in presence of phenyl glycidyl ether^46^.

#### Real Time q-PCR analysis

As seen from the immunoblot analysis, the xenobiotic stress-inducible property of Ylehd could be clearly seen. Whether this expression was regulated at the transcript level could be determined by quantitation of the same upon growing the cells under similar simulated conditions as mentioned above. The RT-qPCR analysis revealed that ylehd RNA transcript levels at 72 h were 30 fold and 26 fold higher in EO and BD grown cells, respectively as compared to those grown only in presence of glucose (Fig. 6B). Thus, it could be said that *ylehd* gene expression was induced in the presence of these model xenobiotic compounds. Hydrolytic conversion brought about by Ylehd could lead to the generation of less toxic alcohols and diols that could be easily assimilated, suggesting that this could be the strategy used by the yeast for adaptation to these compounds. Few such reports on *Y. lipolytica* exist with one report showing induced expression of mitochondrial proteins by the yeast during adaptation to pH stress^47^.

**Figure 7:**
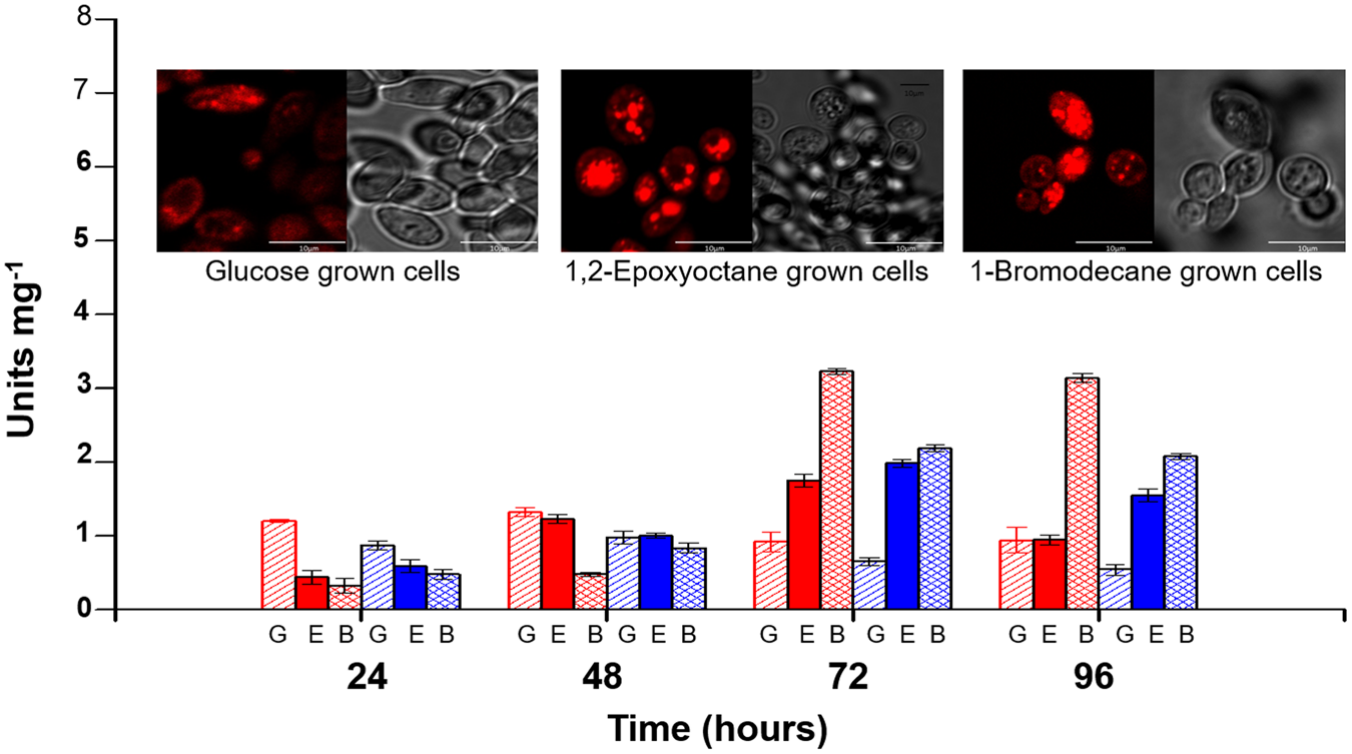
Time course study of total intracellular EH and HLD activity. EH (red) and HLD (blue) activity of *Y. lipolytica* NCIM 3589 grown on Glucose (G), EO (E) and BD (B) for 24, 48, 72 and 96 h. Inset: Nile red staining of cells at 72 h.

Further, the intracellular levels of total EH and HLD activities were also determined as a function of time in the yeast cells grown under simulated marine conditions using the model xenobiotic compounds (Fig. 7). As noted, intracellular levels of total EH and total HLD are much higher at 72 and 96 h in cells grown on EO and BD. The results corroborate the data obtained from protein expression by immunoblot analysis and from quantitation of transcripts by qPCR. However, low activity levels for total EH and HLD could be seen even in glucose grown cells suggesting some constitutive expression. It is therefore likely other isoforms of EH and/or HLD could also be occurring in the yeast cells giving rise to these basal levels. As mentioned earlier we were able to identify 2 other proteins *viz*., XP_499652 and XP_502171 with putative EH activity which could possibly impart these additional activities. The expression of various dehalogenases is regulated and induced by the presence of substrates such as halocarboxylic acids, chloroalkanes, chloroacrylic acids and 1-chlorobenzoate suggesting well developed catabolic pathways. For example, in dichloromethane utilizing methylotrophs, DcmR controls expression of dichloromethane dehalogenase DcmA while in *Rhodococcus erythropolis* the plasmid localized haloalkane dehalogenase gene(dhaA)is regulated by dhaR gene^48^. In contrast, in cases such as *Xanthobacter autotrophicus* GJ10, the expression of HLD is constitutive wherein the addition of 1,2-dichloroethane does not enhance expression and is seen as a sign of evolutionary primitivity^49^. In most microorganisms, EHs are intracellular and constitutively expressed whereas plants are known to contain multiple EH isoforms which are either constitutively expressed or induced by pathogen infections or hormones involved in host defence. In this study, an increase in RNA transcript, protein expression and activity levels of Ylehd in presence of the model xenobiotic compounds is seen, suggesting its induction in their presence.

### Localisation of Ylehd in *Y. lipolytica*

**Figure 8:**
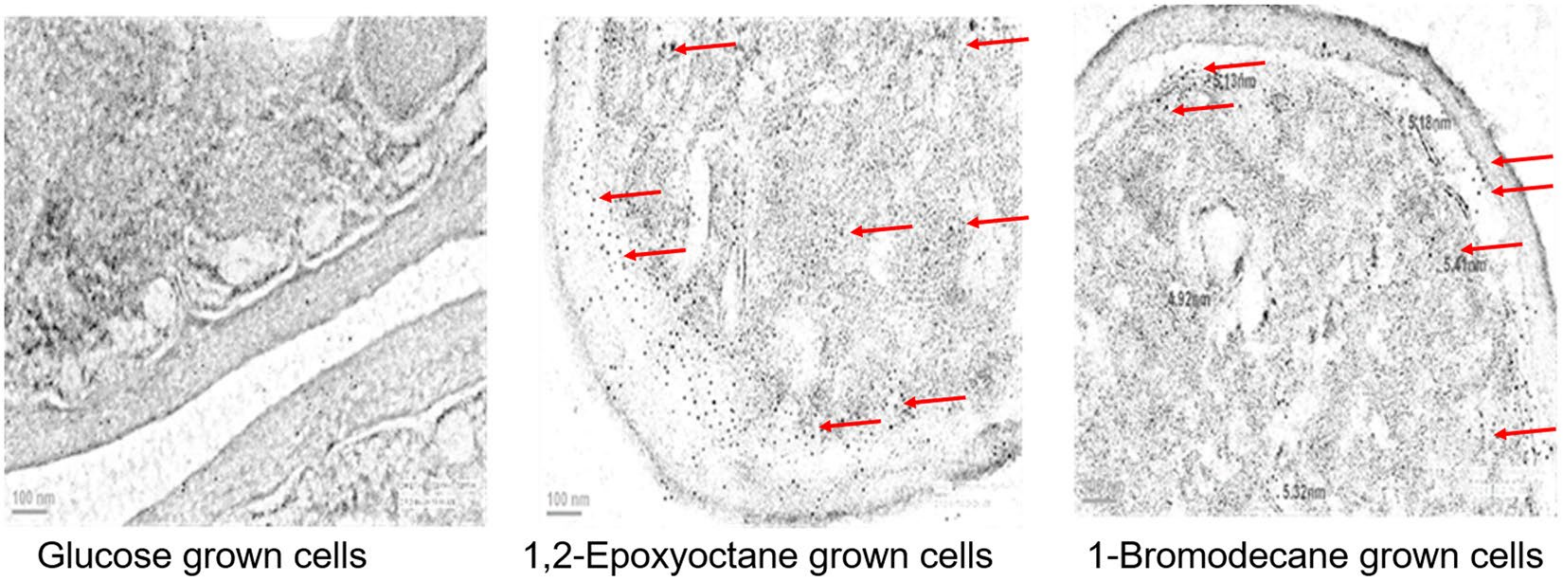
Immunoelectron micrographs for Ylehd expression in *Y. lipolytica* cells. Immunostained sections of cells *Y. lipolytica* cells grown in Glucose, EO and BD at 72 h. Sections stained were viewed at a magnification of 19,500X and colloidal gold nanoparticles of 5 nm observed as shown with arrows.

As yet there is little information available about the subcellular distribution of dehalogenase in microbes. Similarly, though EHs are localized either in the cytosol or microsomal fractions in humans, scarce information is available about their intracellular localisation in microbes. We thus examined the intracellular localisation of Ylehd, in 1.0 µm thick sections of yeast cell with immunoelectron microscopy using the anti-Ylehd primary antibodies and colloidal gold labelled secondary antibodies. Spots in Fig. 8 show Ylehd expression in sections of cells grown on glucose, BD and EO at the end of 72 h. A high density of spots can be seen in EO and BD grown cells as compared to glucose grown ones again emphasizing the enhanced expression of Ylehd in presence of xenobiotics. The gold labelled spots were seen throughout the cytosol and periplasmic space, suggesting localisation of Ylehd.

### Lipid accumulation by *Y. lipolytica*

In our earlier studies, we have suggested a degradative pathway for bromoorganics in *Y. lipolytica* NCIM 3589^21^. The yeast dehalogenates long chain haloalkanes such as BD with the hydrolytic removal of bromide to decanol which is further degraded to a C_10_ fatty acid, decanoic acid and is eventually metabolized to CO_2_. Degradation of EO could be brought about similarly by a hydrolytic step to cleave the epoxide ring releasing the diol which could be metabolized to form neutral lipids as seen in the yeast *Lipomyces* sp^50^. Being a known lipid accumulator, it was hypothesized that if *Y. lipolytica* cells grown on simulated media containing BD and EO are able to detoxify and metabolise the compounds, their degradation should lead tolipid accumulation as shown in our earlier studies. As seen in Fig. 7 (inset), lipid accumulation is seen to a greater extent in BD and EO than in glucose grown cells suggesting the potential of the yeast to detoxify and metabolise these compounds. This study holds relevance as for a microbe to have good bioremediation potential it should not only be able to detoxify the pollutant but also preferably metabolise it.

Fungi are known to actively participate in detoxification and bioremediation by epoxidation and hydroxylation of aromatic or aliphatic pollutants, including polychlorinated dibenzo-*p*-dioxin, alkanes and alkyl-substituted aromatics^51^. They do so by using enzymes such as cytochrome P450 oxygenases, reductases, hyroxylases and dehalogenases. It is to be noted that Ylehd did not possess cytochrome P450 oxygenase or reductase activity. CYP52 proteins from the cytochrome P450 family, reported in *Y. lipolytica* bring about alkane degradation and so far no studies exists of their role in dehalogenation^52^.

In conclusion, *Y. lipolytica* NCIM 3589 isolated from the polluted marine niche would inherently possess the ability to debrominate the natural bromoorganics present in the niche. Over a period of time on exposure to pollutants of anthropogenic origin with varied scaffolds such as epoxides and haloorganics, it is likely to have adapted to them for its survival. Ylehd from *Y. lipolytica* 3589 on functional characterization exhibited epoxide hydrolase and promiscuous haloalkane dehalogenase activity at pH 8.0 and 4.5, respectively and is therefore a likely candidate to help the yeast in its adaptation. Ylehd showed similarities to the α/β hydrolase superfamily with conservation of catalytic triad and HGFP and GFPGS motifs. Such similarities in motifs have suggested that members of this superfamily must have originated from a common ancestor, diverging into proteins with diverse hydrolytic functions depending on the selection pressure in the environment with many members exhibiting multi-functionality^26^. In fact EHs have evolved from gene fusion between N-terminal domain of haloacid dehalogenase and haloalkane dehalogenase. This the first report of a bifunctional EH/HLD from this yeast which is induced in presence of substrates. The clear demarcation of a pH induced toggle in activity is unusual and studies are underway to understand the role of pH switch towards survival under xenobiotic stress. With insights into its pathways and enzyme(s) involved in detoxification and its ability to metabolise bromorganics and epoxides, this yeast would be a good candidate for bioremediation. Such a yeast system with stress inducible proteins could also serve as an efficient biomarker for sensing environmental pollution.

## Materials and Methods

### Strains and media

Medium components and supplements were obtained from Himedia (India). Solvents and compounds were procured from Sisco Research Laboratory (India), Merck (India) or Sigma Aldrich (USA). Molecular biology reagents and kits were sourced from Bangalore Genei (India). *Escherichia coli* DH5α and *E. coli* BL21AI (Invitrogen, USA) were used for clone construction and expression studies, respectively. Both strains of *E. coli* were routinely grown and maintained on Luria Bertani (LB) broth or agar supplemented with 50 µg ml^−1^ Kanamycin when necessary*. Yarrowia lipolytica* NCIM 3589 cells grown for 24 h on YPD medium (gL^−1^): yeast extract, 10.0; peptone, 20.0; dextrose, 20.0 were used for genomic DNA extraction following standard protocol^53^. Simulated sea salts medium(gL^−1^): minimal salt solution media^5^ supplemented with peptone 5; yeast extract 3; potassium dihydrogen phosphate 5; Tween 40 1 ml, was used for studying intracellular expression of Ylehd.

### Sequence identification and analysis

The protein sequences of known HLDs were downloaded from SWISS-PROT database and the consensus sequence obtained as described in Supplementary information Table S1. The putative protein sequence, XP_504164, thus obtained was further analyzed to identify conserved residues aligning, in a multiple sequence alignment, with those of other functionally well characterized EH and HLD proteins as given in Supplementary Table S1. Phylogenetic analysis of Ylehd with the same set of sequences was also carried out in MEGA 6^54^.

### Cloning, expression and purification of Ylehd

The open reading frame (ORF) XM_504164 (hereafter called as *ylehd*) was PCR amplified from *Y. lipolytica* genomic DNA as template and primers as given in Supplementary information. Grown cells were harvested and resuspended in Buffer A: Tris-sulphate, pH 8.0, 50 mM; sodium sulphate, 200 mM; glycerol 5% (v/v) and disrupted by sonication (Labsonic M Sartorius, Germany). The lysate was centrifuged at 16000 g for 30 min and Ylehd purified on Ni-Immobilized Metal Affinity Chromatography resin (Sigma) as per manufacturer’s protocol followed by gel filtration on a Superdex S200 10/300GL (GE Healthcare) equilibrated with buffer A. The homogeneity of the purified protein was ascertained on a 12% native PAGE^53^ and estimated by Bradford method^55^ using bovine serum albumin as standard. Molecular mass of Ylehd was determined by SDS-PAGE. To ascertain the effect of pH, protein was passed through a Superdex S200 10/300GL connected to Akta Prime Plus FPLC machine(GE Healthcare, India). The column had been equilibrated with 50 mM sodium acetate buffer pH 4.5 or 50 mM Tris-sulphate buffer pH 8.0, as the case may be. Column calibration was done with 3–4 mg ml^−1^ proteins of known molecular mass (kDa) *viz*, Ribonuclease A (16.0), Carbonic anhydrase (29.0), Ovalbumin (43.0), Conalbumin (75.0), Aldolase (158.0), Catalase (232.0). Stokes radius of the protein collected at the two pH conditions was calculated using the equation^56^ LogRs = 0.369 log (molecular weight) − 0.254.

### Enzyme assays and product identification

The Epoxide hydrolase (EH) activity was estimated spectrophotometrically by measuring residualEO at 590 nm^57^. EH was added to an assay mixture containing 1 mM of substrate in 50 mM Tris-sulphate, pH 8.0, 2 ug of enzyme and incubated for 30 min at 30 °C. One unit (U) of enzyme activity is defined as the amount of enzyme catalysing the degradation of 1 µmol of EO per min. Product identification for bioconversion of EO to 1,2-octanediol was done by reacting 5 mM EO in 50 mMTris-sulphate, pH 8.0 containing 50 µg of protein under nitrogen-purged conditions in order to eliminate the role of aerial and dissolved oxygen. After 60 min the reaction was stopped by addition of ethyl acetate and concentrated by drying. The product identified by GC-MS using the NIST Mass Spectral Library (NIST05) as mentioned in Supplementary information.

EH activity gel assay was done as follows: to a 1.5% agar solution, EO (100 mM) was added and poured in a petri plate. Native PAGE (12%) loaded with 50 units of enzyme was run under standard non-denaturing, non-reducing conditions at 4 °C. This gel was overlayed on EO agar prepared above and incubated at 30 °C till clearance was seen.

For the dehalogenase assay, to the substrate (5 mM) which was sonicated for 5 minutes in 50 mM sodium acetate buffer, pH 4.5,DTT treated purified enzyme (20–40 µg) was added and incubated at 30 °C for 30 min and halide released measured at 460 nm^58^. One unit (U) of enzyme activity was defined as the amount of enzyme that catalysed the formation of 1 µmol of halide per min. Zymogram carried out by loading protein (20 µg) on 12%native PAGE containing emulsified BD. The activity was visualized by incubating the gel in 50 mM sodium acetate, pH 4.5 and 1 mM DTT at room temperature till grey coloured bands developed^59^. Product identification for HLD was done with BD (5 mM) prepared in 50 mM sodium acetate pH 4.5 at 30 °C. The reaction mix was initially purged with nitrogen for 30 min to remove aerial and dissolved oxygen and initiated by adding 100 µg of protein containing 5 mM DTT and incubated for 60 min. The reactants extracted with diethyl ether and analyzed by GC-MS as mentioned in Supplementary information.

### Biochemical characterization of Ylehd

All experiments involving catalytic activities of the enzyme were performed with BD (5 mM) for HLD and EO (1 mM) for EH activity. The experiments were carried out thrice and values expressed as mean. The best-fit values were achieved by using the software, Microcal Origin 8.0 (Microcal Software Inc., Northampton, USA).

#### Effect of pH and temperature

For temperature optima, EH and HLD assay were performed at temperature of 20–50 °C and assayed as mentioned above. The pH dependence of enzyme for EH and HLD activity was determined in the Britton Robinson buffers with a pH range of 2 to10. The buffer was prepared as a mixture of 0.04 M H_3_BO_3_, 0.04 M H_3_PO_4_ and 0.04 M CH_3_COOH and the desired pH was achieved by titration with 0.2 M NaOH.

#### Substrate specificity and enzyme kinetics

Activity of HLD at pH 4.5 and EH at 8.0 was checked using varioushalogenated (5 mM) and epoxy (1 mM) compounds, respectively as mentioned above and expressed as percent relative activity. Kinetic constants (K_m_, V_max_, k_cat_/K_m_) were determined using Michaelis-Menton and Lineweaver-Burk equations. BD (0.01 to 0.5 mM) and EO (0.5 to 3 mM) were incubated with the enzyme (20 and 2 µg, respectively)for30 min and determined as mentioned above.

#### Effect of additives and metal ions

Ylehd was incubated with 1–5 mM of reducing agents (Dithiothreitol, β-mercaptoethanol and reduced Glutathione) and metal salts such as ferrous sulphate, ferric sulphate, zinc sulphate, nickel sulphate, mercuric sulphate, cobalt nitrate, manganese sulphate, silver nitrate and lead nitratefor 30 min at 4 °C and the percent relative activity for EH and HLD determined as mentioned above.

#### Secondary structure of Ylehd

Secondary structure of Ylehd was determined by circular dichroism spectroscopy. Far uv-CD spectrum was recorded at 30 °C with a Jasco J-810 spectrometer (Jasco, Tokyo, Japan) and measured from 190 to 260 nm with 0.1 mg ml^−1^ protein in 5 mM Tris-sulphate buffer (pH 8.0) and 5 mM sodium acetate buffer pH 4.5. Each spectrum was an average of 10 scans, corrected for absorbance caused by buffer and analyzed using SELCON 3 in Dichroweb http://dichroweb.cryst.bbk.ac.uk/html/home. shtml.

### Expression of Ylehd in whole cells of *Y. lipolytica*

#### EH and HLD activity in lysates of *Y. lipolytica* grown on different carbon sources

*Y. lipolytica* cells were grown in minimal salts medium containing 1.0% glucose and grown at 30 °C for 22 hours, washed by centrifugation and re-suspended in water. Using this as a pre-inoculum, three flasks of simulated sea salts medium adjusted to pH 6.5, each containing 5 mM BD, 5 mM EO and 1.0% glucose were grown up to 96 hours. Cells were harvested at differing time points and processed as mentioned in Supplementary information. Equal amount of protein from each lysate was used for checking EH and HLD activity at pH 8.0 and 4.5, respectively.

#### Raising antibodies, titre determination and partial purification

The purified Ylehd protein was used to raise polyclonal anti-Ylehd antibody in rabbits under the Research Proposal Number 54_14–15 and were raised as given in Supplementary information. Western blot analyses using the anti-Ylehd antibody were carried out to measure the levels of Ylehd and was also used to detect the localization of Ylehd in the yeast as mentioned below.

#### Immunoblot analysis

The expression of Ylehd in *Y. lipolytica* cells was carried out usingWestern blot followed by immunodetection. Equal protein from each lysate was run on 12% SDS-PAGE gel and protein bands electro-transferred to nitrocellulose membrane for immunodetection, as per standard protocol^53^. In brief, purified polyclonal antibody 1:100 dilution as the primary antibody while 1:50,000 dilution of the secondary antibody conjugated to horse radish peroxidase(MP Biomedical, U.S.A.) was used. Chemiluminiscence substrate kit (Takara Bio Inc, Japan) was used for developing the bands, visualized and imaged on a C-Digit Blot Scanner from Li-Cor (Nebraska, U.S.A.).

#### Immunoelectron Microscopy

Cells grown under the above mentioned condition for 72 h made free of culture medium by washing in a centrifuge (1000 rpm, 5 min) in 0.1 M Phosphate buffer pH 7.4 and processed for immunoelectron microscopy as mentioned in Supplementary information. Cells were observed under a Morgagni 268D/Tecnai G^2^ 20 Transmission Electron Microscope (Fei Company, The Netherlands) at suitable magnifications (19500x).

#### Real time q-PCR analysis

Total RNA was isolated from *Y. lipolytica* cells, grown for 72 h in medium individually containing 5 mM BD, 5 mM EO and 1% glucose, using Trizol reagent (Thermofisher Scientific, U.S.A.) as per the manufacturers protocol. The detailed protocol is mentioned in Supplementary information. The results obtained were normalized to actin gene expression and analyzed using the ddCT method.

#### Nile Red staining

Visualization of lipid bodies in intact cells was done by Nile red (1 mg ml^−1^ solution in acetone, Sigma Aldrich, California, U.S.A.) staining, wherein, the stain was added to the cell suspension (1/10, vol/vol) and incubated for 1 hour at room temperature. Cells were harvested, washed twice with distilled water, and re-suspended in 50 mM sodium phosphate buffer, pH 6.0 to an *A*_600_ of 2.5. Imaging was performed with a Nikon C1Si confocal laser scanning microscope (Nikon, Japan).

### Data Availability

All data generated and, analysed during this study are included in this manuscript as well as in its Supplementary Information.

### Statistical Data Analysis

All the experiments have been carried out in 3 separate sets where every set has been replicated thrice. The data is expressed as average ± SEM (Standard Error of Mean). ANOVA (Analysis of variance) was used and all values for *p* that were ≤0.05 were considered significant.

## Electronic supplementary material

Ylehd, an epoxide hydrolase with promiscuous haloalkane dehalogenase activity from tropical marine yeast Yarrowia lipolytica is induced upon xenobiotic stress
