# Supplementary material for: Ylehd, an epoxide hydrolase with promiscuous haloalkane dehalogenase activity from tropical marine yeast Yarrowia lipolytica is induced upon xenobiotic stress
Source: Sci Rep. 2017 Sep 19;7:11887. doi: 10.1038/s41598-017-12284-9 (PMC5605520; doi:10.1038/s41598-017-12284-9)
Supplement: Supplementary file 1 — Ylehd, an epoxide hydrolase with promiscuous haloalkane dehalogenase activity from tropical marine yeast Yarrowia lipolytica is induced upon xenobiotic stress [file 41598_2017_12284_MOESM1_ESM.pdf]

Supplementary Information

Ylehd, an epoxide hydrolase with promiscuous haloalkane dehalogenase activity from tropical marine yeast *Yarrowia lipolytica* is induced upon xenobiotic stress

Authors: Chandrika Bendigiri, Smita Zinjarde, Ameeta RaviKumar

Supplementary Results

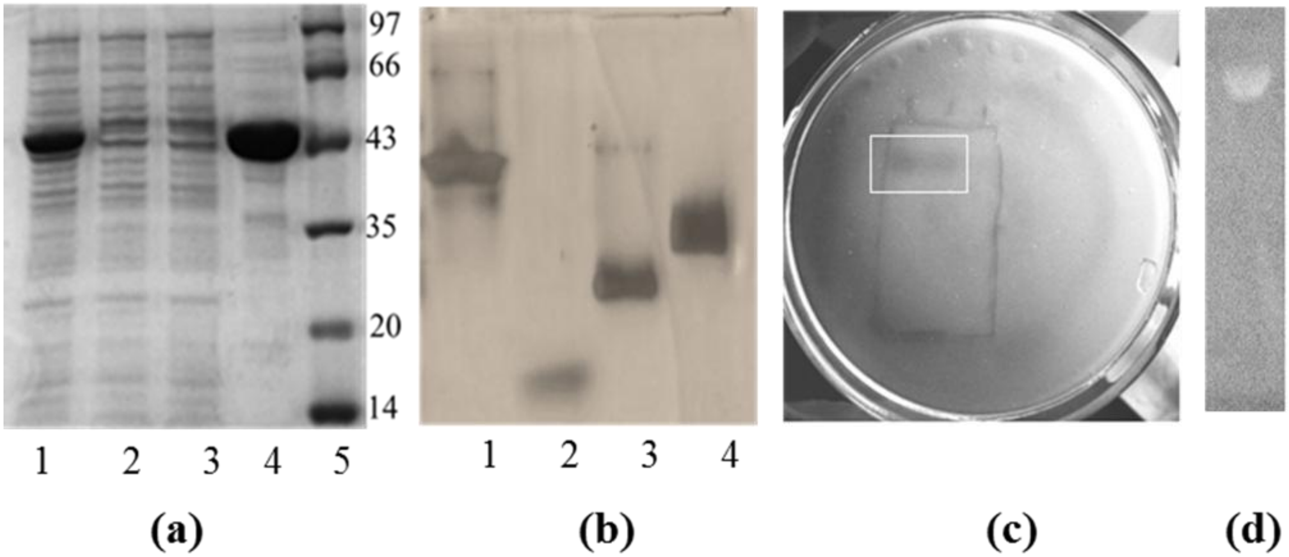

Supplementary Figure S1: Protein purification and activity gel

(a) SDS-PAGE (12%) of expressed Ylehd on Ni-NTA column: sample before loading (lane1), flow through from Ni-NTA column (lane 2); wash (lane 3); elute from Ni-NTA column (lane 4): Protein molecular weight markers (lane 5).

(b) Native PAGE (12%) of purified protein: Elute from Supedex 200 size exclusion chromatography (lane 1); standard Ovalbumin 43 kDa (lane 2); standard Bovine serum albumin 66 kDa (lane 3); standard Conalbumin 75 kDa (lane 4.)

(c) Activity staining for EH with the rectangle showing the zone of clearance.

(d) Activity staining for HLD with the grey coloured band.

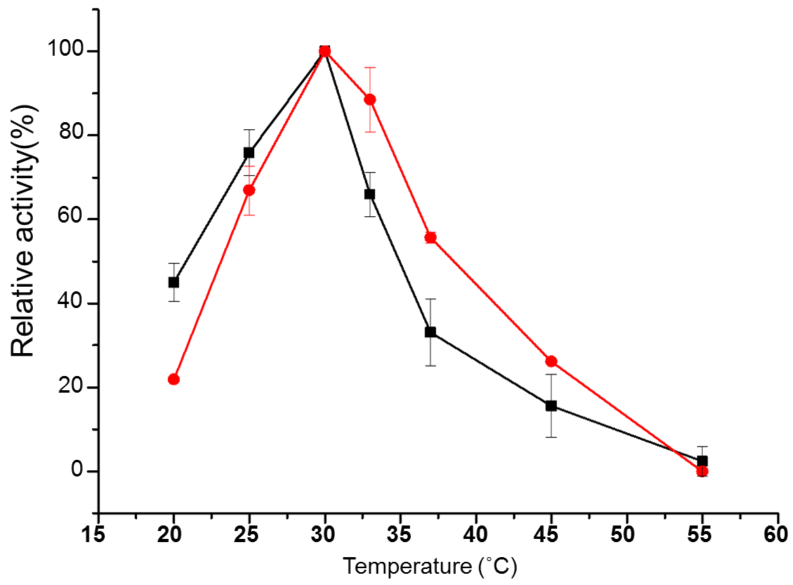

Supplementary Figure S2: Temperature optimum for Ylehd

EH(black) and HLD(red) activity of Ylehd doe at temperatures ranging from 20-50˚C**.**

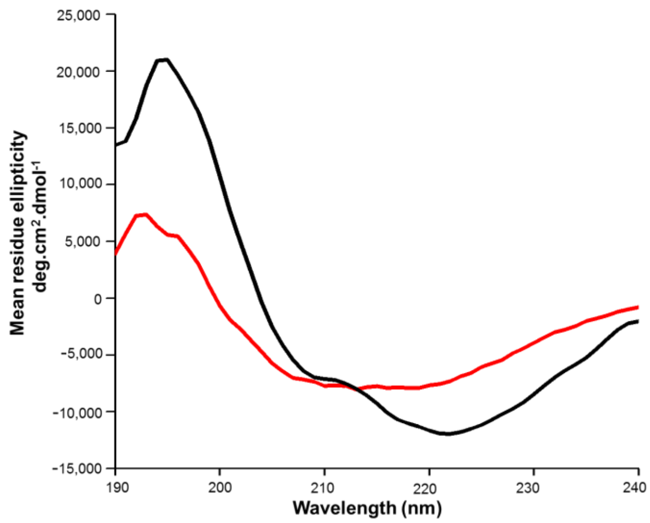

Supplementary Figure S3: Far-UV circular dichroism spectra of Ylehd

Spectra at pH 8.0 (black) and at pH 4.5 (red)

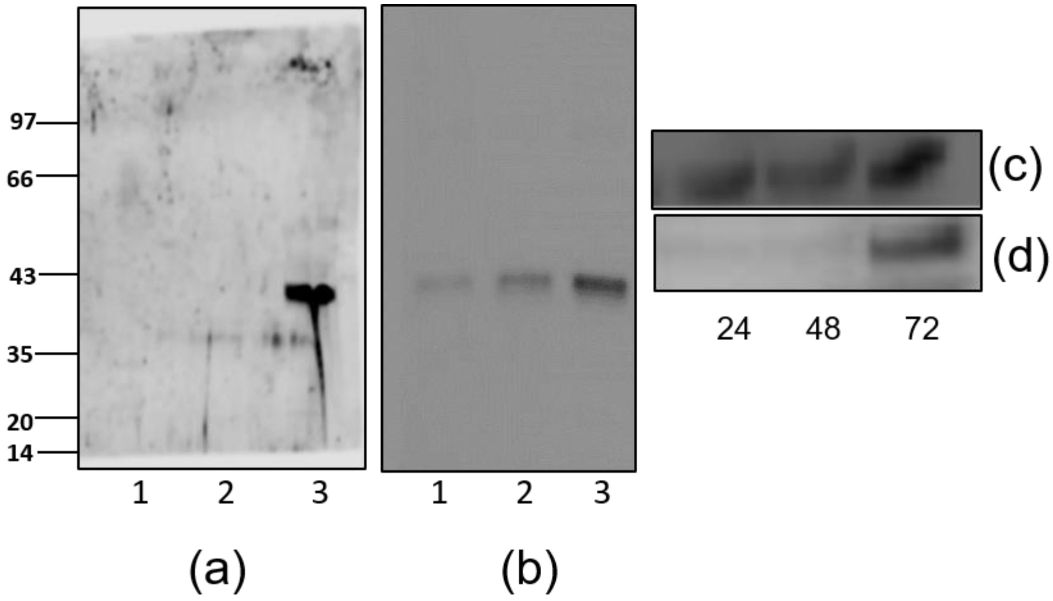

Supplementary Figure S4: Western blots

Full blots of Ylehd in (a)EO and (b)BD grown cells with lanes 1,2 and 3 representing cells grown and lysed after 24, 48 and 72 hours respectively (cropped images shown in Figure 5).

Additional exposures for (c) BD and (d) EO grown cells similarly after 24, 48 and 72 hours

Supplementary Table S1: Sequence information of proteins used for phylogenetic analysis

Name of protein	Activity	*Name of organism*	NCBI accession number[a] or Uniprot id[b]
DbjA	HLD	*Bradyrhizobium japonicum* USDA 110	NP_7677271[b]
DbeA	HLD	*Bradyrhizobium elkani* USDA 94	AB478942 [b]
DhaA	HLD	*Rhodococcus rhodochrous* NCIMB 13064	Q53042 [a]
DspA	HLD	*Strongylocentrotus purpuratus*	XP_794172 [b]
DadB	HLD	*Alcanivorax dieselolei*	YP_006819020.1 [b]
LinB	HLD	*Sphingomonas paucimobilis*	P51698 [a]
DmbA	HLD	*Mycobacterium bovis 5033/66*	AJ7842721 [b]
Dat_A	HLD	*Agrobacterium radiobacter C58*	Q8U671 [a]
DrbA	HLD	*Rhodopirellula baltica SH1*	Q7U599 [a]
DmbC	HLD	*Mycobacterium bovis 5033/66*	A4Q9R7
DhlA	HLD	*Xanthobacter autotrophicus GJ10*	P22643 [a]
Dppa	HLD	*Plesoecystis pacifica SIR-1*	WP_006972606 [b]
DhmA	HLD	*Mycobacterium avium N85*	Q93K00 [a]
DmbB	HLD	*Mycobacterium bovis 5033/66*	AJ7842732 [b]
O31243_RHIRD	EH	*Agrobacterium radiobacter*	O31243 [a]
StEH1	EH	*Solanum tuberosum*	Q41413 [a]
HYES_CORS2	EH	*Corynebacterium sp*	O52866 [a]
BNSEH1	EH	*Brassica napus*	Q8L5G6 [a]
EPH1	EH	*Phaphia rhodozyma*	Q9UUP8 [a]
SPEH1	EH	*Strongylocentrotus purpuratus*	B2MWN2 [a]
HYES_PIG	EH	*Sus scrofa*	Q6Q2C2 [a]
EPXH2	EH	*Homo sapiens*	P34913 [a]
HYES_RAT	EH	*Rattus norvegicus*	P80299 [a]
M_EPXH2	EH	*Mus musculus*	P34914 [a]
SACCK	EH	*Saccharomyces cereviseae*	P53750 [a]
Q9P8X4_RHOGU	EH	*Rhodotorula glutinis*	Q9P8X4 [a]
HYl1	EH	*Aspergillus niger*	Q9UR30 [a]
Ylehd(This study)	EH	*Yarrowia lipolytica*	Q6C598 [a]

Supplementary Table S2: Effect of additives on EH and HLD activities of Ylehd

Property	Epoxide hydrolase[a]	Haloalkane dehalogenase[a]
EDTA	100	100
Reduced Glutathione	100	210
Dithiothreitol	100	200
β mercaptoethanol	100	150
Cu^{2+}	10	NA
Hg^{2+}	NA	NA
Mg^{2+}	95	95
Mn^{2+}	15	NA
Fe^{2+}	85	NA
Fe^{3+}	95	NA
Ag^{2+}	2	6
Ni^{2+}	75	NA
Co^{2+}	NA	10
Pb^{2+}	NA	NA
Zn^{2+}	80	NA
Ca^{2+}	30	36

[a] Enzyme activity expressed as percent relative activity with untreated protein considered as 100%.

NA: No activity seen under the given assay conditions

Supplementary Materials and Methods

Sequence identification and analysis

The protein sequences of known and well characterised HLDs were downloaded from SWISS-PROT database and a multiple sequence alignment (MSA) constructed in CLUSTAL W (https://www.ebi.ac.uk/Tools/msa/ clustalw2). This alignment was then loaded on Matlab tool (Mathworks, USA) to obtain the consensus sequence which was searched against *Y.lipolytica* non-redundant protein sequence database using BLAST-P (http://blast.ncbi.nlm.nih.gov).The putative protein sequence, XP_504164, thus obtained was further used for analysis.

Cloning, expression and purification of Ylehd

The open reading frame (ORF) XM_504164 (hereafter called as *ylehd*) was PCR amplified from *Y.lipolytica* genomic DNA as template and primers (Forward-NheI 5'ATCGTAGCTAGCATGGCGCACCTGACCAAAG-3' and Reverse-XhoI 5'-GTCATCTCGAGCTACAGCTTACTAGTCTTG-3') by using the program: 95°C (5 min), 30 cycles 95°C (45 s),60°C (30 s),72°C (60 s), 72°C (10 min) and 4°C till further use. Amplified *ylehd* was cloned into pET28a vector (Novagen, USA) and confirmed by sequencing on an automated sequencer (3730 DNA Analyzer, Applied Biosystems, USA).The plasmid DNA from *E.coli* transformants was isolated using the plasmid purification kit (Bangalore Genei, India). The transformed *E.coli*BL21AI containing the *ylehd* gene was grown to an $OD_{600\ of}$ 0.6 at 30°C and protein expression induced with 0.8 mM Isopropyl thiogalactopyranoside and 0.05 mM Arabinose for 14 h.

GC-MS conditions for product identification

For EH, the sample containing the product was injected (split as 1:10) on a DB624 column (30m×0.25mm×0.25µm, Agilent, USA) connected to MS (Agilent, USA) using nitrogen as carrier gas. The analysis was done as per the following program: initial temperature at 60°C held for 30 s, rise of 15ºC/min up to 120°C, held for 30 s and further increase of 30°C/min up to 240°C and held for 5 min.

For HLD, the product was identified by split injecting (1:10) it on a Stabilwax column (30 m × 0.25 mm × 0.25 µm, Restek, USA) connected to a GC-MS (Agilent, USA) using nitrogen as the carrier gas. The analysis was done as per the following program: initial temperature of the column was at 60°C, held for 2 min, increased at the rate 10°C/min up to 180°C, held for 1min and increased at 15°C/min up to 200°C and held for 5 min. Identification of the products was done using the NIST Mass Spectral Library (NIST05)

EH and HLD activity in *Y.lipolytica* grown on different carbon sources

Cells were harvested at 24, 48, 72 and 96 h, washed in water and re-suspended in Y-PER (Thermo-Fisher, Pune, India) lysis buffer at a density of 2g wet weight per 5 ml of lysis buffer. The cell suspension was kept under rocking conditions at room temperature for 20 min. Complete lysis of cells as determined microscopically, was achieved by mechanical shearing using acid washed glass beads and vortexing under cold conditions for 5 min. Lysate was centrifuged at 10000xg for 20 min at 4˚C and dialyzed against buffer containing 50mMTris pH 8.0 and 200mM Na_2SO_4. The dialyzed lysate solution was estimated for protein concentration using the Folin Lowry method[1]. Equal amount of protein from each lysate was used for checking EH and HLD activity.

Raising antibodies, titer determination and partial purification

Ylehd protein (500μg) was cleaved off the 6XHis tag (Thrombin Cleavage kit, Sigma Aldrich, California, U.S.A) and re-suspended in 1X PBS. Two healthy female rabbits were selected for immunization and pre-immune serum collected on day of immunization. Rabbits were injected subcutaneously with antigen emulsified in Fruend's Complete Adjuvant. On day 15, rabbits were injected subcutaneously with 500 μg of antigen emulsified in Fruend's Incomplete Adjuvant. On day 21, rabbits were injected intraperitoneally with 500μg antigen in saline. Blood was withdrawn from the ear vein of each rabbit to check antibody titre on day 25. Serum antibody titre was checked by ELISA method. In brief, 10 $\mu g ml^{-1}$ Ag diluted in carbonate buffer were coated on 96 well ELISA plate and incubated overnight at 4˚C. Next, the plate was washed 3 times with physiological saline containing 0.05%Tween 20 (PBST) and 100 μl of 1% BSA in PBST was added in each well and incubated for 1 h at 37˚C. The plate was then washed 4-5 times with PBST and 100 μl of rabbit immune serum (1:100 dilution) and pre-immune serum (1:100 dilution) was added and incubated for 3 h at 37˚C and the plate washed 5 times with PBST. The secondary antibody, namely, Goat anti rabbit IgG HRP (Bangalore Genei, Bangalore India) at 1:1,000 dilution was added (100 μl) and incubated for 1h at 37˚C. The plate was washed 5 times with PBST and 100 μl of 2, 2′-Azino-bis (3-ethylbenzothiazoline-6-sulfonic acid) diammonium salt (ABTS, Sigma, California, U.S.A.) substrate was added and after 10 minutes incubation at 37˚C and the absorbance determined at 405 nm by ELISA reader. After appropriate titre was reached, the animals were bled through ear vein and serum separated and stored in

-20˚C deep freezer till further use.

For antibody purification, 35 ml of the above serum, provided by was concentrated to 10 ml using concentrators (Amicon, Millipore) fitted with 10 kDa cut off membrane, under Nitrogen gas pressure of 1.5 bars. The concentrate was subjected to 60% ammonium sulphate precipitation with constant stirring. The entire solution was allowed to rest for 4 h, centrifuged at 10000 rpm for 20 minutes and pellet re-suspended in minimum quantity of 1XPBS with total volume was now approximately 6ml after resuspension. This was frozen at -20˚C till further use. For further purification the solution was subjected to Protein A agarose chromatography using a kit provided by Bangalore Genei, Bangalore, India. Steps were followed as per the supplier's protocol. Purity of the antibody was ascertained by native gel electrophoresis.

Immunoelectron Microscopy

The yeast cell pellet was dispersed and fixed in 0.2% glutaraldehyde and 4% para-formaldehyde in 50mM phosphate buffer pH 7.4 for 1h at room temperature. Cell suspension was centrifuged (5 minutes) and fixative discarded and washed in0.1 M Phosphate buffer pH 7.4. Cells were again fixed in 4% paraformaldehyde at 4˚C for 2 h. Fixed cells were centrifuged and the supernatant discarded and the pellet washed and centrifuged. The sample pellets were submitted in 0.1 M Phosphate buffer pH 7.4 to TEM Facility, All Institute of Medical Sciences, New Delhi, India. Samples were first dehydrated in an ascending grade of ethanol, infiltrated and embedded in LR white resin (TAAB Laboratories Equipment Ltd., England, UK) at 55˚C. Firstly 1 µm thick sections were cut with an ultramicrotome (Ultramicrotome Leica EM UC7, Wetzlar,

Germany), mounted on to glass slides, stained with aqueous toluidine blue and observed under a light microscope for gross observation of the area. For immuno electron microscopy sections of 80-90 nm thickness were cut and mounted onto 300 mesh nickel grids. The grids were initially incubated in 1% fish gelatin made in 0.01 M phosphate buffer saline (PBS) for 30 minutes for blocking of nonspecific reactions. Following this the grids were incubated in primary antibody (1:50 dilution of rabbit polyclonal purified antibody) overnight at 4°C, using in 0.01 M Phosphate buffer pH 7.4 and 1% fish gelatin as diluent. Next, after a brief wash (1 minute x 3 changes) in diluent buffer, the grids were incubated in secondary antibody (1: 100 dilution of goat anti-rabbit-whole molecule, Sigma Aldrich, California, U.S.A.) conjugated with 5 nm colloidal gold for 2 hours at room temperature. Grids were then washed in buffer and finally in distilled water (one minute each). These were stained with uranyl acetate for 5 minutes, briefly rinsed in distilled water, blotted dry and observed under a Morgagni 268D/Tecnai G^2 20 Transmission Electron Microscope (Fei Company, The Netherlands) at a suitable magnification (7500 to 29000x).

Real time PCR analysis

Total RNA was isolated from *Y.lipolytica* cells, grown for 72 h in medium individually containing 5mM BD, 5mM EO and 1% glucose, using Trizol reagent (Thermofisher Scientific, U.S.A) as per the manufacturers protocol. All samples were quantified using Qubit® RNA BR (Broad-Range) Assay Kit with the Qubit® 2.0 Fluorimeter (Life Technologies USA). cDNA synthesis was carried out using total RNA, Oligo (dT 20 mer 10 mM), dNTPs (10mM), 5X first strand synthesis buffer, DTT, MLV-RT (Reverse

Transcriptase) from Life Technologies, USA and nuclease free water. Primers were designed for the gene of interest and the house keeping gene Actin (XM_502566.2) with sequences; XM_504164: Forward-AGGAGTTTGAGGAGAACGAAGAT, Reverse – ATGTTCAGCTTCTTGAGCAGTTC and XM_502566.2: Forward - GGCCAGCCATATCGAGTCGCA, Reverse -TCCAGGCCGTCCTCTCCC. Real time PCR was carried out using SYBR green mix (TAKARA Biosciences) on the Applied Biosystems StepOne Plus real time PCR instrument. Following cycling parameters: 2 min incubation at 95 ˚C, followed by 35 cycles of 30 s at 94 ˚C, 30 s at 55 ˚C, and 30 s at 72 ˚C. Melting curves were obtained under the default settings of the instrument. Results were normalized to actin gene expression and analyzed using the ddCT method[2].

Supplementary references

1. Lowry, O. H., Rosebrough, N. J., Farr, A. L. & Randall, R. J. Protein Measurement with the Folin Phenol Reagent. *J. Biol. Chem.***193,** 265–275 (1951).
2. Livak, K. J. & Schmittgen, T. D. Analysis of relative gene expression data using real-time quantitative PCR and the 2(-Delta DeltaC (T)) Method. *Methods San Diego Calif* **25,** 402–408 (2001).
